# Comparative proteomics profiling revealed the involvement of GRB2‐ROCK2 axis in Lyme neuroborreliosis caused by *Borrelia Burgdorferi*

**DOI:** 10.1111/jcmm.17253

**Published:** 2022-02-25

**Authors:** Yunfeng Bi, Jianjun Liu, Mingbiao Ma, Lvyan Tao, Yun Peng, Xiting Dai, Zhenhua Ji, Ruolan Bai, Miaomiao Jian, Taigui Chen, Lisha Luo, Feng Wang, Zhe Ding, Aihua Liu, Fukai Bao

**Affiliations:** ^1^ Yunnan Province Key Laboratory for Tropical Infectious Diseases in Universities Kunming Medical University Kunming China; ^2^ Department of Dermatology the Second Affiliated Hospital of Kunming Medical University Kunming China; ^3^ Yunnan Key Laboratory of Stem Cell and Regenerative Medicine Biomedical Engineering Research Center Kunming Medical University Kunming China

**Keywords:** *Borrelia burgdorferi*, chemokine pathway, GRB2, Lyme neuroborreliosis, proteomics, ROCK2

## Abstract

The zoonotic Lyme neuroborreliosis (LNB) disease is caused by *Borrelia burgdorferi*, with wide distribution, rapid dissemination and high disability rate. However, the molecular mechanism underlying *B*. *burgdorferi* mediated neuroborreliosis remains largely unknown. Here, the frontal cortex from rhesus brains was incubated with *B*. *burgdorferi*, and proteomics profiling was evaluated by isobaric tag for relative and absolute quantitation. Proteins were identified and quantified, and differentially expressed proteins (DEPs) were isolated by comparing co‐cultured samples and control samples. A total of 43, 164 and 368 DEPs were significantly altered after 6, 12 and 24 h treatment with *B*. *burgdorferi* respectively. Gene ontology and KEGG pathway analyses revealed that chemokine biological process was significantly enriched. Two genes in chemokine pathway including GRB2 and ROCK2 were significantly up‐regulated after *B*. *burgdorferi* co‐culturing. By in vitro assay, we confirmed that the expression of GRB2 and ROCK2 was increased after *B*. *burgdorferi* infection. In conclusion, our study revealed the involvement of chemokine pathway in the pathogenesis of LNB. GRB2 and ROCK2 may be novel biomarkers and therapeutic targets for LNB.

## 1 INTRODUCTION

Lyme disease is the most common tick‐borne illness in America and Europe, and is caused by the spirochete *Borrelia burgdorferi* (*Bb*). Lyme Neuroborreliosis (LNB) occurs in about 15% of patients with untreated *Bb* infection.^1^, ^2^, ^3^, ^4^ *Bb* is highly neurotropic and can be latent in central or peripheral nervous system for a long time. Bb causes different neuropathies at different stages. For instance, central nervous system lesions mostly manifest memory impairment caused by encephalitis. Peripheral nervous system lesions mostly manifest as abnormal sensation, hemiplegia and spasm. Therefore, understanding the pathogenesis of LNB is of great significance for the prevention and treatment of Lyme disease.

Accumulating evidence demonstrates that LNB is a chronic inflammation disorder of central nervous system (CNS).^2^, ^5^, ^6^ Chemokines secreted by astrocytes play multiple roles in the pathogenesis of chronic inflammation disorder of CNS.^7^, ^8^, ^9^ In order to elucidate molecular mechanism of LNB, it is urgent to reveal the relationship between regional immune characteristics of brain tissue and LNB, and identify new therapeutic targets.

Rhesus are recognized as experimental animal models that can accurately simulate human LNB. Therefore, the establishment of rhesus monkey as LNB animal model has important clinical significance. In this study, frontal cortex of rhesus monkeys was co‐cultured with spirochetes in vitro. Based on proteomic profiling analysis by isobaric tag for relative and absolute quantitation (iTRAQ), we identified differentially expressed proteins (DEPs), and further confirmed the role of chemokine pathway in the pathogenesis of LNB.

## 2 METHODS

### 2.1 Materials

Three healthy rhesus macaques (one‐year‐old, two males and 1 female) with no any infection were obtained from the Institute of Medical Biology, Chinese Academy of Medical Sciences and Peking Union Medical College (animal permit number: SCXK (DIAN) K2015‐0004). The euthanasia procedure was performed according to the rules for euthanasia of experimental animals (http://www.lascn.net/), the Guide for the Care and Use of Laboratory Animals,^10^ and the ARRIVE Guidelines for Reporting Animal Research.^11^ This experiment was reviewed and approved by the Animal Ethical and Welfare Committee of Kunming Medical University.

### 2.2 Spirochetes culture

*Borrelia burgdorferi* standard 4680 strain was purchased from American Type Culture Collection and grown in Barbour‐Stoenner‐Kelly II (BSK II) medium supplemented with 6% rabbit serum (Thermo Fisher Scientific, Carlsbad, CA, USA) according to the methods described previously.^12^ The cultures were incubated at 37°C under microaerophilic conditions till late logarithmic phase. The cells were collected by centrifugation (4°C, 10 min, 2000 × g), washed twice with sterile PBS, and adjusted to a final concentration of 1 × 10^7^ bacteria/mL with an appropriate volume of RPMI 1640 medium supplemented with 10% foetal bovine serum (FBS, Thermo Fisher Scientific).

### 2.3 Frontal cortex brain explant co‐culture with spirochetes

Frontal cortex brain explant was treated with spirochetes as previously reported.^13^ Briefly, freshly harvested frontal cortex tissues were collected at necropsy and washed twice with PBS. Then, the frontal cortex was sliced, and each sliced section was placed in a T‐25 flask (Corning, NY) containing 4 mL of 10% FBS‐RPMI 1640 medium. Triplicate tissue sections were then incubated in a humidified incubator with 5% CO_2_ at 37°C for 6, 12 and 24 h, respectively. After incubation, the co‐cultured frontal cortex brain sections were collected and stored at −80°C.

### 2.4 Protein extraction and 8‐plex iTRAQ labelling

Individually and co‐cultured frontal cortex brain samples were ground in liquid nitrogen. The total proteins were then extracted in a solution containing 200 ul of L3 buffer (50 mM Tris‐Cl, pH8, 8 M urea, 2 M thiourea, 2 M EDTA, 1 × protease inhibitors cocktails), 800 μl of ice‐cold acetone and 10 mM dithiothreitol. The concentration of total protein was determined using the Bradford assay. Subsequently, an aliquot of 100 μ g of protein from each sample was reduced, alkylated and digested with trypsin according to the manufacturer's protocol (Applied Biosystems, Framingham, MA). The peptides were iTRAQ labelled as shown in Table 1, mixed and vacuum dried.

**TABLE 1:** iTRAQ labelling

Sample ID	Batch ID	Label ID
P_6h−1	Batch 1	113
Bb_6h−1		114
P_12h−1		115
Bb_12h−1		116
P_24h−1		117
Bb_24h−1		118
P_6h−2	Batch 2	113
Bb_6h−2		114
P_12h−2		115
Bb_12h−2		116
P_24h−2		117
Bb_24h−2		118

### 2.5 2D‐RPLC fractionation and MS/MS analysis

LC‐MS/MS (Triple TOF 5600 plus, AB SCIEX, Framingham, USA) analysis was performed as described previously.^14^ Briefly, the iTRAQ labelled peptide mixture was reconstituted in reverse phase liquid chromatography (RPLC), and then, fractionated through a high pHC18 high‐performance liquid chromatography system (Dinoex Ultimate 3000 BioRS, Thermo Fisher) equipped with Durashell‐C18 reverse phase column. Eluted fractions were monitored by determining the absorbance at 214 nm wavelength. Each fraction was desalted with a Strata X C18 column (Phenomenex, Torrance, CA), vacuum dried and reconstituted in 15 μl of buffer (0.1% formic acid, 2% acetonitrile in Milli‐Q water). Fractions were separated by a 90 min linear gradient of 5% to 76.4% acetonitrile in 0.1% formic acid at a flow rate of 350 nl/min. The MS scan was set as 350–1250 m/z for 0.25 s scanning. Subsequently, MS/MS scans were performed for 0.04 s under a spray voltage of 2300 V. The dynamic exclusion for MS/MS was 12 s. The MIAPE reporting guidelines were followed.^15^

### 2.6 Protein identification and quantification

Protein identification and quantification were performed using ProteinPilot (v4.0.8085) software.^16^ Parameters for Proteinpilot search were listed in Table 2. The raw data were searched against the UniProtKB database.^17^ To minimize false discovery rate, the confident value was set as ≥95% (amount to the confident value “unused ProtScore” ≥ 1.3 in ProteinPilot software), and the unique peptide was set as one.^16^ Fold change was defined as up‐regulated (>1.5) and down‐regulated (<0.67), and *p* < 0.05 was set to identify differentially expressed proteins (DEPs).

**TABLE 2:** Parameters for Protein pilot search

Item	Value
Type of search	iTRAQ 8‐plex (Peptide Labelled)
Enzyme	Trypsin
Cys alkylation	Iodoacetamide
Instrument	TripleTOF 5600
Bias correction	TRUE
Background correction	TRUE
ID focus	Biological modifications
Search effort	Thorough ID
Protein mass	Unrestricted
Database	Uniprot Macacamulatta20171231.fasta (45,199 items)

### 2.7 GO and KEGG enrichment analysis

Gene Ontology (GO) and KEGG enrichments were conducted according to the methods described previously.^16^, ^18^ Briefly, the Database for Annotation, Visualization and Integrated Discovery (DAVID) database (https://david.ncifcrf.gov/) were utilized, and DEPs were mapped into DAVID to identify the biological processes, cellular components, and molecular function of DEPs. The map of the KEGG pathway was obtained from the KEGG database (https://www.genome.jp/kegg/pathway.html), and DEPs were analysed for the enrichment of KEGG pathways. Fisher's exact test was used to identify the significantly enriched GO terms or KEGG pathways. *p* < 0.05 was considered as the threshold for significant enrichment.

#### 2.7.1 Real‐time PCR

Real‐time PCR was performed as described previously.^13^ Primer sequences were as follows: GRB2 5′‐TAGCAAACAGCGGCACGAT‐3′ and 5′‐TAGACGTTCCGGTTCACG‐3′; ROCK2 5′‐GGTTACTATGGGCGAGAA‐3′and 5′‐TTTAGGAATTGGGAAGGT‐3′; β‐actin 5′‐AGGCTCTCTTCCAACCTTCCTT‐3′ and 5′‐CGTACAGGTCTTTACGGATGTCCA‐3′. The cycling parameters were initial denature at 95°C for 30 s, and then, 40 cycles of 95°C for 5 s and 60°C for 30 s. The relative mRNA expression was calculated by 2^−△△CT^ method with β‐actin as internal control.

#### 2.7.2 Western blotting

Western blotting was performed as described previously,^19^ using GRB2 primary antibody (Abcam, ab32037), ROCK2 primary antibody (Abcam, ab125025) and goat anti‐rabbit secondary antibody labelled with horseradish peroxidase (Abcam, ab6721).

#### 2.7.3 Statistical analysis

Statistical analyses were performed using the software package GraphPad PRISM version 7 (GraphPad Software, Inc.),^20^ and unpaired two‐tail Student's *t*‐test was used to evaluate the *p*‐values. *p* < 0.05 was considered statistically significant.

## 3 RESULTS

### 3.1 Overview of experimental design and analytical strategy

**FIGURE 1:**
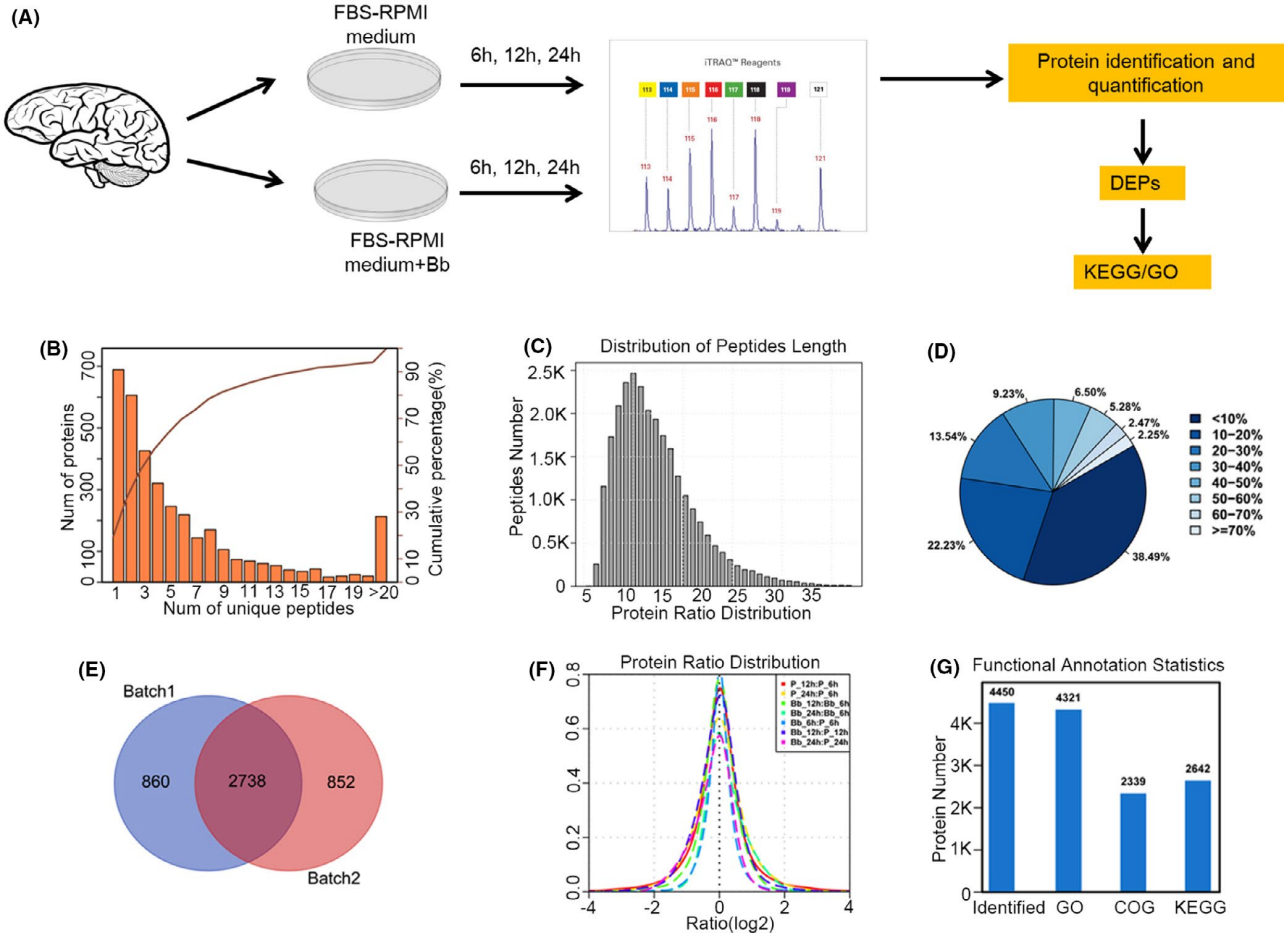
Overview of experimental design, analytical strategy and data assessment. (A) Sections of Rhesus frontal cortex were co‐cultured with *Bb* in vitro. Control group was set by culturing frontal cortex with RPMI 1640 medium supplemented with 10% FBS. Samples were harvested after 6, 12 and 24 h of co‐culturing. Proteins were extracted for iTRAQ. Protein identification and quantification were determined with ProteinPilot software. Differentially expressed proteins (DEPs) and related biological pathways were identified. (B) Distribution of the number of proteins with different number of unique peptides. (C) Length distribution of peptide segment. (D) Pie plot showing the different percentage of protein coverage distribution

To uncover the molecular basis of LNB caused by *Bb* infection, we established the monkey brain in vitro infection model by co‐culturing rhesus frontal cortex with *Bb* in vitro. Control group was set by culturing frontal cortex with foetal bovine serum. Two batch of specimens under treatment of three time points (6, 12 and 24 h) were collected and iTRAQ technique was performed for protein profiling for each sample. We performed protein identification by using Proteinpilot (V4.5), a search engine companied with AB Sciex 5600,^21^ with the parameters shown in Table 2. We further filtered the protein list derived from Proteinpilot with the unused score >1.3 and containing at least one unique peptide. The identified peptide segments were further filtered with confidence >95. Then, DEPs were determined with student's *t*‐test and biological enriched pathway were derived from web‐accessible database DAVID. The overall experimental design and data process were shown in Figure 1A. Statistical summary such as distribution of unique peptide segment number (Figure 1B), length distribution of peptide segment (Figure 1C) and protein coverage distribution (Figure 1D) were also provided.

DEPs were defined based on fold change (up‐regulated > 1.5 and down‐regulated < 0.67), with *p* < 0.05 indicating significant difference. Finally, we identified 43, 164 and 368 DEPs after 6, 12 and 24 h co‐culturing respectively.

### 3.2 Chemotaxis pathway was significantly affected in brain tissue co‐cultured with *Bb*

**FIGURE 2:**
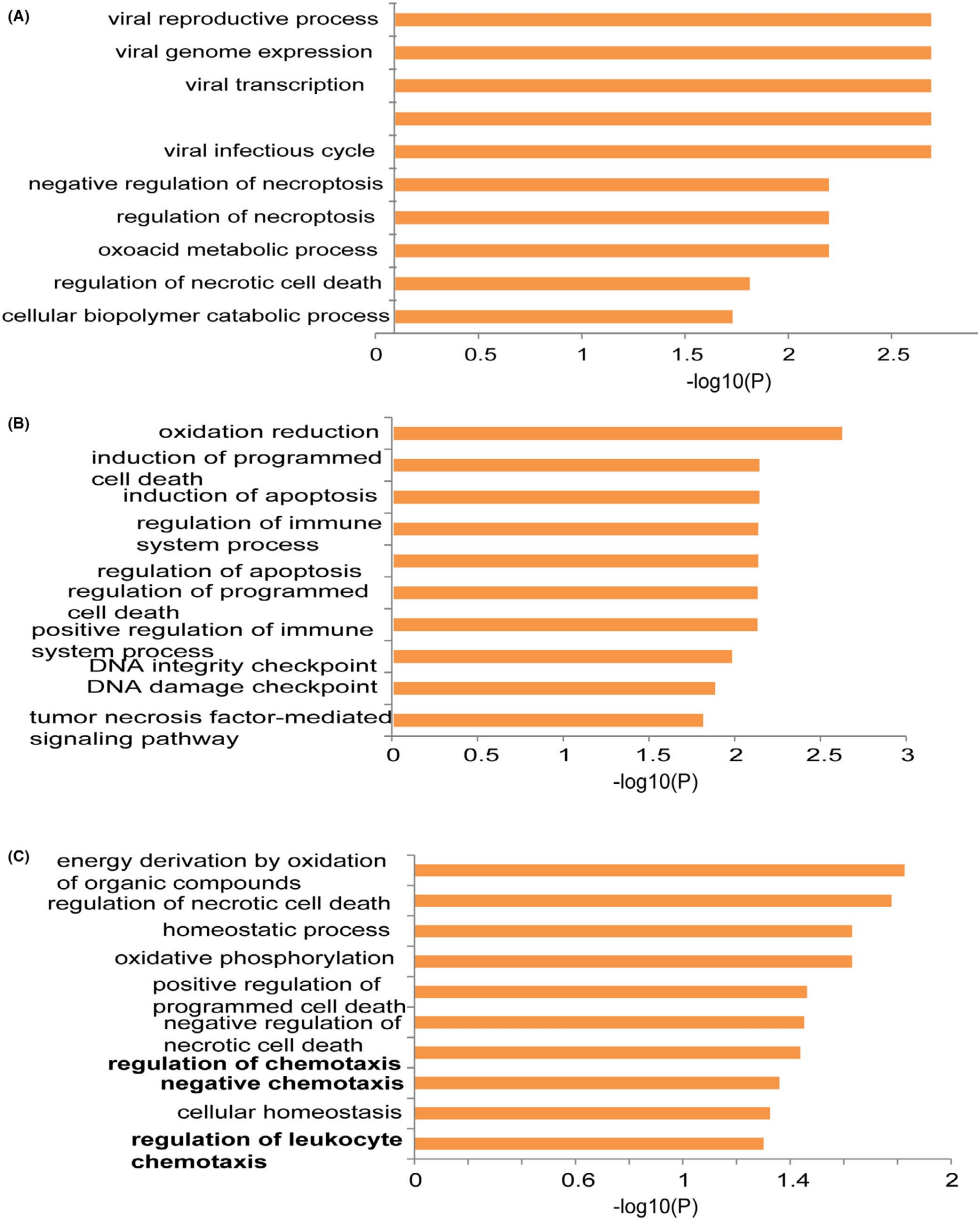
GO analyses of DEPs at different time points after co‐culture. (A) At 6 h after co‐culture, DEPs were significantly enriched in viral infection related pathways, necrotic cell death and oxoacide metabolic process biological pathways. (B) At 12 h after co‐culture, biological pathways such as oxidation reduction, programmed cell death, apoptosis and DNA checkpoint were significantly enriched. (C) At 24 h after co‐culture, oxidative phosphorylation, programmed cell death, necrotic cell death and chemotaxis biological pathways were significantly enriched for DEPs

To explore biological processes in frontal cortex co‐cultured with Bb, we performed GO term enrichment analysis of DEPs. GO comprises three orthogonal ontologies, including molecular function (MF), biological process (BP) and cellular component (CC). Our results showed that DEPs at 6 h were significantly enriched in viral infection related pathways, necrotic cell death and oxoacide metabolic process biological pathways (Figure 2A). Biological pathways, such as oxidation reduction, programmed cell death, apoptosis and DNA checkpoint, were significantly enriched at 12 h treatment (Figure 2B). At 24 h treatment, oxidative phosphorylation, programmed cell death, necrotic cell death and chemotaxis biological pathways were significantly enriched for DEPs (Figure 2C). These results confirmed that biological pathways, such as chemotaxis pathway, were remarkably affected in brain tissue co‐cultured with Bb.

### 3.3 KEGG pathway analysis of DEPs

**FIGURE 3:**
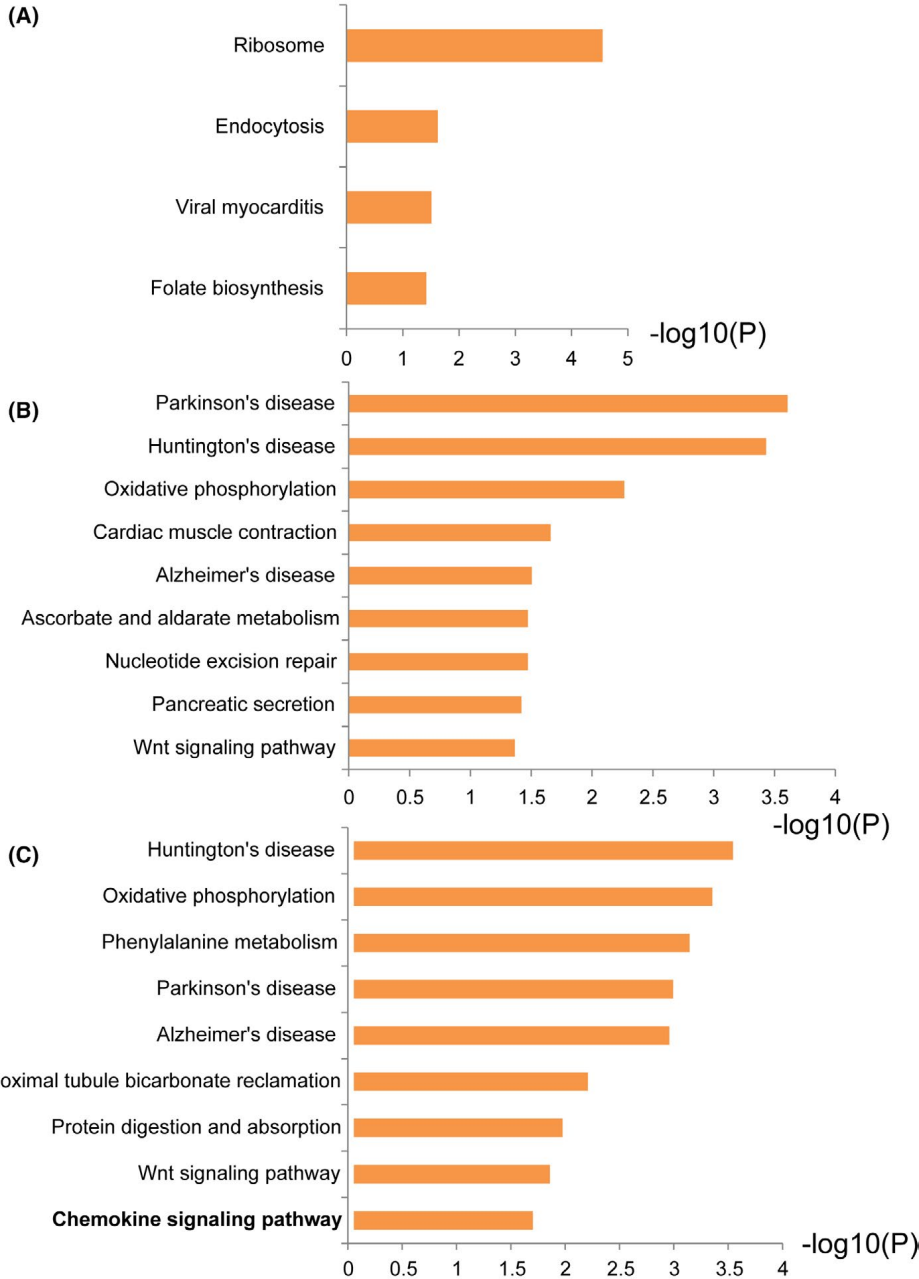
KEGG analyses of DEPs at different time points after co‐culture. (A) At 6 h after co‐culture, ribosome, endocytosis, viral myocarditis and folate biosynthesis pathways were significantly enriched. (B) At 12 h after co‐culture, neurodegenerative disorders including Parkinson's disease, Huntington's disease, Alzheimer's disease were enriched. Oxidative phosphorylation and Wnt signalling pathway were also enriched. (C) B. At 24 h after co‐culture, neurodegenerative diseases pathways, oxidative phosphorylation and chemokine signalling pathway were enriched

KEGG mapping is the process to map genes or proteins to molecular interaction, reaction and relation networks. Next, we performed KEGG pathway analysis of DEPs at 6, 12 and 24 h of time points. The results showed that ribosome, endocytosis, viral myocarditis and folate biosynthesis pathways were significantly enriched at 6 h treatment (Figure 3A). Neurodegenerative disorders including Parkinson's disease, Huntington's disease, Alzheimer's disease were enriched at 12 h treatment (Figure 3B). Oxidative phosphorylation and Wnt signalling pathway were also enriched. Neurodegenerative diseases pathways were also enriched at 24 h treatment, consistent with disease phenotype (Figure 3C). Oxidative phosphorylation and chemokine signalling pathway were also enriched at 24 h treatment.

**FIGURE 4:**
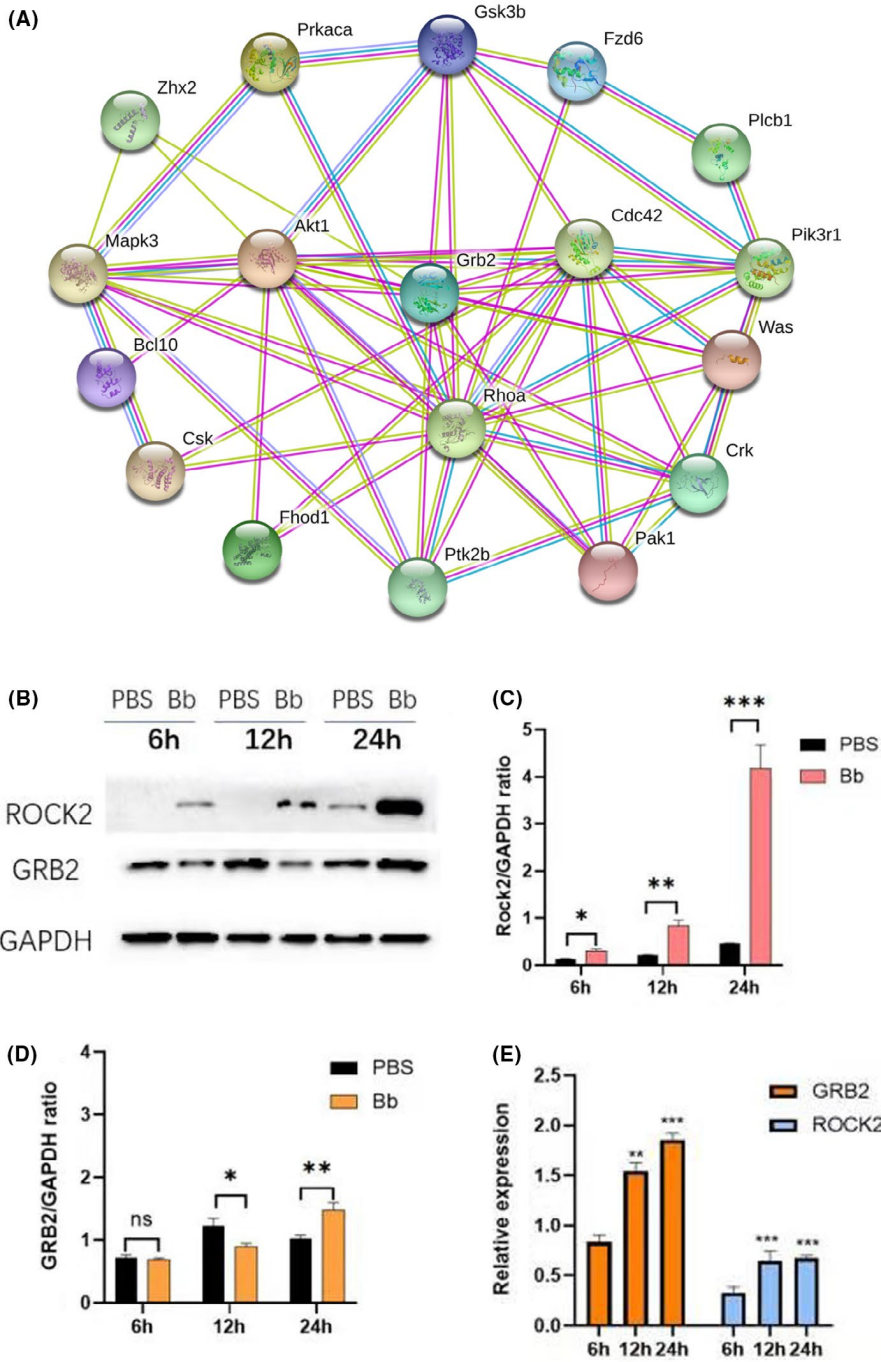
PPI network and expression analysis of ROCK2 and GRB2. (A) PPI network analysis of GRB2 and ROCK2. (B) Western blot analysis of ROCK2 and GRB2 after 6, 12 and 24 h of Bb infection. Shown were representative bolts from three independent experiments with new samples. (C) Densitometry analysis of ROCK2 protein levels. *p < 0.05, **p < 0.01, ***p < 0.001. *N* = 3. (D) Densitometry analysis of GRB2 protein levels. ns: not significant, *p < 0.05; **p < 0.01. *N* = 3. E. Real‐time PCR analysis of GRB2 and ROCK2 mRNA levels after 6, 12 and 24 h of Bb infection. Data were from three independent experiments with new samples. **p < 0.01, ***p < 0.001. *N* = 3

To further investigate the role of chemokine signalling pathway in Bb infection, we checked the expression of proteins in this pathway among different treatment groups (Table 3). GRB2 expression decreased at 12 h treatment but significantly increased at 24 h treatment. PPI network analysis showed that GRB2 was highly associated with other protein in the chemokine pathway (Figure 4A). We confirmed the upregulation of GRB2 and ROCK2 at both protein and mRNA levels during *Bb* co‐culturing by Western blotting (Figure 4B,C,D) and real‐time PCR (Figure 4E).

**TABLE 3:** Changes of expression of the proteins in chemokine pathway

Protein name	6 h	12 h	24 h
	FC and *p*‐value	FC and *p*‐value	FC and *p*‐value
AC	0.941	1.010	1.067
	0.579	0.874	0.258
Akt	0.934	0.533	1.570
	0.403	0.060	0.346
Cdc42	1.014	0.537	1.770
	0.742	0.133	0.780
CrK	0.830	1.793	1.295
	0.417	0.230	0.068
CSK	1.132	0.636	0.626
	0.988	0.396	0.504
ERK1	1.065	1.240	3.294
	0.584	0.344	0.101
Gαi	1.043	1.433	11.262
	0.323	0.049	0.034
Gβγ	2.260	1.686	2.871
	0.287	0.073	0.008
GRB2	1.003	0.526	9.810
	1.000	0.028	0.024
GRK	1.065	1.170	0.934
	0.584	0.556	0.735
GSK3B	1.122	0.677	0.671
	0.651	0.445	0.213
IKK	0.801	1.110	0.634
	0.235	0.940	0.126
MAPK3	1.083	0.906	0.671
	0.366	0.551	0.254
PAK1	1.189	0.916	0.946
	0.245	0.164	0.647
PI3K	1.220	1.107	0.598
	0.131	0.870	0.324
PKA	1.087	1.057	1.108
	0.205	0.205	0.318
PKC	0.871	2.006	2.681
	0.623	0.029	0.111
PLCβ	0.820	1.249	7.949
	0.044	0.156	0.011
PTK2B	0.834	0.733	0.649
	0.371	0.349	0.282
Rac	1.507	0.682	0.484
	0.156	0.023	0.203
Raf	0.863	1.672	0.921
	0.607	0.253	0.671
Rap1	0.756	0.383	0.418
	0.472	0.194	0.332
RhoA	1.194	0.563	0.428
	0.534	0.036	0.111
ROCK	0.943	1.570	4.018
	0.782	0.136	0.044
SOS	0.962	0.853	0.710
	0.821	0.258	0.126
WASP	0.646	0.803	0.618
	0.197	0.573	0.482
β‐arrestin	0.852	0.959	1.011
	0.444	0.070	0.895

Note

The proteins in Bb group were up‐regulated by >1.2) and down‐regulated by <0.83, compared with control group. For *p* < 0.05, the expression of protein in Bb group was regarded as significantly different. The differences between two batches were averaged.

## 4 DISCUSSION

Lyme disease is transmitted to human through the bite of infected blacklegged ticks. LNB occurs in up to 15% of patients with untreated Bb infection. However, the pathogenesis of LNB is not fully understood. Inflammation and innate immune response, which is triggered by the effort of the host to eliminate the pathogen, is known as the first line of defence against pathogens. In present study, we attempted to characterize the interaction between the host and the bacterium in the CNS via the co‐cultivation of the frontal cortex of rhesus macaques and Bb by proteomics profiling. We have shown distinct alterations of proteomics in the frontal cortex brain by co‐culturing with Bb. We identified that several biological pathways, such as chemokine signalling pathway and several neurodegenerative diseases, were overrepresented.^22^ Among the proteins in chemokine signalling pathway, GRB2 is a scaffold protein involved in propagating signalling by growth factor and cytokine receptors, such as epidermal growth factor receptor (EGFR). The SH2 domain of Grb2 can bind to phosphorylated tyrosine residues on EGFR, while SH3 domain can interact with proline‐rich regions of other proteins, such as Son of Sevenless. Diseases associated with GRB2 include Wiskott‐Aldrich syndrome and hepatitis.^23^ GRB2 has been reported to be associated with chemokine.^24^ ROCK2 has been reported to be involved in inflammation regulation.^25^, ^26^ For example, oxymatrine activated RhoA/ROCK2 pathway, leading to acute intestinal inflammation.^25^ In addition, vitamin D inhibited inflammatory response by suppressing RhoA/ROCK2/NF‐ĸB pathway.^26^ Therefore, both GRB2 and ROCK2 are important regulators of inflammation.

Elucidation of inflammatory responses to *Bb* during persistent dissemination in non‐human primate model can better understand the pathogenesis of LNB, compared with rodent models.^27^, ^28^ The glia cells can trigger the innate immune responses and inflammation during *Bb* infection by recruiting peripheral immune cells and releasing chemokines and cytokines.^29^, ^30^ The neurological sequelae is accompanied by the apoptosis of glial cells and neurons.^31^ In particular, chemokine CXCL13 has been proposed as a potential biomarker for LNB.^32^ Consistent with these observations, our current study demonstrated that the DEPs related to chemokine pathway were markedly increased during infection with Bb, such as IL6ST, CCL24CX3CR1, CSF1R and C1QTNF7. Meanwhile, the complement and coagulation cascades that can regulate immune response and inflammation were still active 24 h after Bb infection. While our recent integrative transcriptome and proteome analysis revealed a complex interaction network between Bb and frontal cortex explants of Rhesus,^13^ in this study, we focused on chemokine pathway and further confirmed that GRB2 and ROCK2, two key regulators of chemokine pathway, were up‐regulated at both protein and mRNA levels by Bb infection in Rhesus brain.

In conclusion, based on proteomics profiling of Rhesus brain exposed to Bb, we revealed that chemokine pathway was significantly overrepresented after Bb infection. In particular, we confirmed that the upregulation of two key proteins of chemokine pathway GRB2 and ROCK2 was associated with the pathogenesis of LNB. Further functional studies are necessary to demonstrate the role of chemokine pathway in the pathogenesis of LNB and validate GRB2 and ROCK2 as novel biomarkers and therapeutic targets for LNB.

## 5 ETHICS STATEMENT

The study was reviewed and approved by the Animal Ethical and Welfare Committee of KMU. Approval for the animal experimentation was granted by the Guide for the Care and Use of Laboratory Animals and the ARRIVE Guidelines for Reporting Animal Research.

## CONFLICT OF INTEREST STATEMENT

The authors declare no conflict of interest.

## AUTHOR CONTRIBUTIONS

Fukai Bao, Aihua Liu and Yunfeng Bi designed the experiments. Yunfeng Bi, Jianjun Liu, Mingbiao Ma, Lvyan Tao, Yun Peng, Xiting Dai, Zhenhua Ji, Ruolan Bai, Miaomiao Jian, Taigui Chen, Lisha Luo, Feng Wang and Zhe Ding performed the experiments. Fukai Bao, Yunfeng Bi and Jianjun Liu analysed the data. Fukai Bao and Yunfeng Bi wrote the paper. All authors read and approved the final manuscript.

## AUTHORS CONTRIBUTIONS

**FUkai Bao:** Conceptualization (lead); Data curation (lead); Resources (lead); Supervision (lead); Visualization (lead); Writing – review & editing (lead). **Yunfeng Bi:** Conceptualization (lead); Data curation (lead); Formal analysis (lead); Funding acquisition (lead); Investigation (lead); Methodology (lead); Project administration (lead); Resources (lead); Software (lead); Supervision (lead); Validation (lead); Visualization (lead); Writing – original draft (lead); Writing – review & editing (lead). **Jianjun Liu:** Funding acquisition (lead); Investigation (lead); Methodology (lead); Software (lead); Supervision (lead). **Mingbiao Ma:** Methodology (equal). **Lvyan Tao:** Methodology (equal). **Yun Peng:** Methodology (equal). **Xiting Dai:** Methodology (equal). **Zhenhua Ji:** Methodology (equal). **Ruolan Bai:** Methodology (equal). **Miaomiao Jian:** Methodology (equal). **Taigui Chen:** Methodology (equal). **Lisha Luo:** Methodology (equal). **Feng Wang:** Methodology (equal). **Zhe Ding:** Methodology (equal). **aihua liu:** Conceptualization (equal).

## Data Availability

All original data and the datasets generated for this study are available on request to the corresponding author.
